# Supplementary material for: Tumor matrix stiffness promotes metastatic cancer cell interaction with the endothelium
Source: EMBO J. 2017 Jul 10;36(16):2373–89. doi: 10.15252/embj.201694912 (PMC5556271; doi:10.15252/embj.201694912)

Appendix table of contents

Appendix Supplementary Figure Legends S1-S6

Appendix Supplementary Methods

Appendix Supplementary Figures

Supplementary figure legends

Appendix Figure S1. CCN1 is upregulated by stiffness and controls N-Cadherin levels

A Representative western blot analysis showing CCN1 abundance in the conditioned medium, extracellular matrix (ECM) and harvested cells (intracellular) after culture on FN-coated PAGs. HUVECs were cultured on PAGs for 7 days to generate a layer of ECM before cells were removed and the matrix collected for western blot analysis. GAPDH was used as loading control for the intracellular fraction.

B HMVECs pre-treated with blebbistatin were seeded on FN-coated PAGs with different stiffness for 24 h in the presence of blebbistatin. Representative western blot showing CCN1 and N-Cadherin (CDH2) levels. GAPDH was used as loading control.

C Representative image of in situ hybridization for Ccn1 of E0771 orthotopic tumor showing the heterogeneous expression of Ccn1 within the tumor, where high Ccn1 expression is found in peri-necrotic areas (necrosis highlighted within the blue line). Scale bars = 100 µm.

Appendix Figure S2. CCN1 affects β-catenin activity and N-Cadherin expression

A Representative western blot analysis showing that silencing β-catenin (siCTNNB1) in HUVECs blocks the increase in N-Cadherin (CDH2) levels induced when HUVECs are cultured on FN-coated PAGs of high stiffness. Quant = CDH2 intensity over GAPDH intensity, which was used as loading control. Analysis by Image Studio Lite software.

B Immunofluorescence staining showing the nuclear localization of active β-catenin (unphosphorylated on residues S37/T41). Scale bar = 100 µm.

C Representative western blot analysis showing the transfection efficiency of CCN1-GFP in HUVECs used in Figure 4F. GAPDH was used as loading control.

D Representative western blot analysis for total β-catenin showing the silencing efficiency of β-catenin in HUVECs overexpressing CCN1-GFP. GAPDH was used as loading control.

Appendix Figure S3. CCN1 controls N-Cadherin levels and endothelial-cancer cell binding

A Immunofluorescence staining for N-Cadherin (CDH2) and VE-Cadherin (CDH5) showing that VE-Cadherin abundance and localization is not altered when HUVECs knocked down for CCN1 are cultured on FN-coated PAGs of different stiffnesses. Conversely, N-Cadherin levels decrease when CCN1 is knocked down. White arrowheads highlight localization of CDH2 at the cell periphery. Scale bar = 20 μm.

B RT-PCR showing the knockdown efficiency of CCN1 in HUVECs used in panel (A) and Figure 5A. Bars = mean +/- SEM (n = 3 technical replicates).

C Silencing of CCN1 in HUVECs does not influence the permeability of these cells cultured as a monolayer on a transwell, as assessed by FITC-dextran permeability. Bars = mean +/- SEM. N siCTL = 5, n siCCN1 = 6 measurements from 2 independent experiments. ns = not significant based on two-tailed unpaired t-test.

D Representative western blot showing the knockdown efficiency of CCN1 in HUVECs used in Figure 5B.

E Representative western blot showing the knockdown efficiency of CCN1 in HUVECs used in Figure 5C.

F Representative western blot showing N-Cadherin expression in E0771 breast cancer and Lewis Lung Carcinoma (LLC) cells.

G Representative western blot showing the knockdown efficiency of CCN1 in HMVECs used in Figure 5D.

H Representative western blot showing the knockdown efficiency of β-catenin (CTNNB1) in HUVECs used in Figure 5E.

I Transfection efficiency of CCN1-GFP in HUVECs used for Figure 5F.

J Representative western blot analysis showing N-Cadherin protein levels in PC3 cells infected with lentiviral vector expressing shRNA targeting CDH2 used in Figure 5G.

K Representative western blot analysis showing that immortalized human mammary normal fibroblasts (iNF) or cancer associated fibroblasts (iCAF) similarly increase CCN1 levels with matrix stiffness.

L Representative western blot analysis showing that the knockdown of CCN1 in iNFs, iCAFs or PC3 cells similarly decreases CDH2 levels.

M Western blot analysis showing CCN1 protein levels of PC3 cells infected with lentiviral vector expressing shRNA targeting CCN1 used in Figure 5G.

N Representative western blot analysis showing CCN1 protein levels in PC3 cells silenced for CCN1 with siRNA and used for the experiment in (O).

O Silencing of CCN1 with a pool of siRNA in PC3 cells reduces their binding to a monolayer of HUVECs. Bar = Mean +/- SEM (n = 3 replicate experiments). P-value based on two-tailed unpaired t-test.

For western blot, GAPDH was used as loading control.

Appendix Figure S4. HTNC treatment of B16F10 transplanted *Rosa26*$^{flSTOP\text{-}tdRFP}$ mice induces recombination in the tumor vasculature.

A MS analysis of isolated mouse lung endothelial cells showing that Ccn1 protein levels are reduced when treated with HTNC (1 µg/ml) in culture for 16 h.

B Representative immunohistochemistry for RFP (from one of two mice that were treated with HTNC and that showed positive staining using anti-RFP antibody) showing positive staining in tumor blood vessels and lung of HTNC treated *Rosa26*$^{flSTOP\text{-}tdRFP}$ B16F10 tumor bearing mice, and absence of staining in BSA treated (CTL) mice. Scale bar = 100 µm. Arrowheads highlight RFP positive cells.

C Pecam1 and RFP staining quantification in tumor and lung tissue of one *Rosa26*$^{flSTOP\text{-}tdRFP}$ mouse treated with HTNC (B). RFP staining shows that HTNC induced recombination in some regions of the tumor and lung. Pecam1 staining quantification was used to show the total amount of blood vessels in the two tissues. n = number of ~250 μm^2 regions of the tumor/lung tissue assessed in one analyzed tissue section.

D Flow cytometry analysis of blood and bone marrow cell subpopulations isolated from HTNC or BSA treated *Rosa26*$^{flSTOP\text{-}tdRFP}$ B16F10 tumor bearing mice, showing the absence of recombination in these cells. For the blood, representative plots of RFP expression in circulating $Ly6G^+CD11b^+$ neutrophils, $Ly6C^+CD115^+$ monocytes and Lineage positive ($CD3^+$, $CD19^+$, $NKp46^+$, $Ter119^+$) cells. For the bone marrow, representative plots of RFP expression in bone marrow $Ly6G^+CD11b^+$ neutrophils, $Ly6C^+CD115^+$ monocytes and Lineage positive ($CD3^+$, $CD19^+$, $NKp46^+$, $Ter119^+$) cells. Data represent the mean ± S.D. of RFP^+ cells for each sample. Neutrophils gated on: FSC-SSC, single cells, live cells, $lineage^-$, $Ly6G^+$, $CD11b^+$, $CD115^-$. Monocytes gated on: FSC-SSC, single cells, live cells, $lineage^-$, $Ly6G^-$, $CD11b^+$, $CD115^+$, $Ly6C^+$. $Lineage^+$ cells gated on: FSC-SSC, single cells, live cells, $lineage^+$. RFP^+ CTL = Cells isolated from Rosa-$CreER^+$ mouse, as positive control. Data represent the mean +/- S.D. of RFP^+ cells for each sample (n = 2 mice).

E Representative image of Ccn1 (left, in situ hybridization) and Pecam1 (right) staining in a blood vessel of B16F10 tumor. Consecutive tissue sections. Scale bar = 100 µm.

Appendix Figure S5. HTNC treatment of B16F10 transplanted *Ccn1*$^{Plox/Plox}$ mice induces recombination in the tumor vasculature but does not affect tumor vascularity.

A B16F10 tumor growth was not affected by HTNC treatment. Bars = mean +/- SEM. N = 7 mice for each treatment group.

B Representative immunofluorescence staining of (C) and (D). Scale bar = 100 µm.

C, D B16F10 tumor vascularity (C) and pericyte coverage (D) was not affected by HTNC treatment as indicated by quantification of Meca32 (endothelial cells) staining and colocalization of Meca32 and NG2 (pericytes), respectively. Bars = Mean +/- SEM. N = mice assessed.

E Representative immunostaining for pimonidazole adduct showing hypoxic areas in the tumors quantified in (F).

F Tumor hypoxia as indicated by pimonidazole staining of tissue sections was not affected by HTNC treatment. N = mice assessed.

G Flow cytometry analysis showing that there were no significant differences in GFP^+ B16F10 melanoma cells found in the lungs of *Ccn1 WT* and *Ccn1* KO^{EC} mice after 24h from intravenous injection. N = mice assessed.

H,I Endothelial conditional KO of Ccn1 (*Ccn1* KO^{EC}) did not reduce the formation of metastasis in the lungs, as shown by lack of differences in lung weight (normalized by total mouse weight, H) and number of metastasis (I) between *Ccn1 WT* and *Ccn1* KO^{EC} mice injected tail vein with B16F10 cells. N = mice assessed after two weeks from i.v. injection.

Significance according to the two-tailed unpaired t-test. ns = not significant.

Appendix Figure S6. Silencing of endothelial CCN1 reduced cancer cell transendothelial migration.

A Western blot analysis showing the efficiency of CCN1 silencing, and N-Cadherin (CDH2) downregulation, in HUVECs used in Figure 7A,B.

B Western blot analysis showing the efficiency of CCN1 silencing, and N-Cadherin downregulation, in HMVECs used in Figure 7C,D.

C Silencing of CCN1 in HMVEC does not influence the permeability of the cells cultured in a monolayer on a transwell, as assessed by FITC-dextran permeability. Bars = mean +/- SEM (n siCTL = 19, n siCCN1 = 13 fields assessed from 4 independent experiments).

Appendix Supplementary Methods

MS analysis

Digested peptides were injected on an EASY-nLC system coupled on line to a LTQ-Orbitrap Elite via a nanoelectrospray ion source (Thermo Scientific). Peptides were separated using a 20 cm fused silica emitter (New Objective) packed in house with reversed-phase Reprosil Pur Basic 1.9 µm (Dr. Maisch GmbH) and eluted with a flow of 200 nl/min from 5% to 30% of buffer containing 80% ACN in 0.5% acetic acid, in a 90 min linear gradient (for in-gel digested peptides) or 190 min gradient (for SAX fractions). The mass range acquired for the full MS scan was 300-1,650 m/z with a resolution of 240,000 (rCID mode) or 120,000 (HCD mode) at 400 Th and the Orbitrap aimed to collet 1 x 10^6 charges at a time. For HCD fragmentation, the top ten most intense peaks in the full MS were isolated for fragmentation with a target of 5,000 (rCID mode) or 40,000 ions at a resolution of 15,000 at 400 Th (HCD mode). Ions singly charged were excluded, whilst the ions that have been isolated for MS/MS were subsequently added to an exclusion list. MS data were acquired using the Xcalibur software (Thermo Scientific) and .RAW files processed with the MaxQuant computational platform (Cox & Mann, 2008) and searched with the Andromeda search engine(Cox et al, 2011) against the human UniProt (2010) database (release-2012 01, 88,847 entries). MaxQuant version 1.4.1.6 was used for the stiffness and CCN1 knockdown proteomes, whilst 1.3.6.0 and 1.4.1.0 were used for the contact inhibited and Matrigel proliferation datasets, respectively. MaxQuant was run

with the following settings: To search the parent mass and fragment ions we required a mass deviation of 4.5 ppm and 20 ppm (HCD) or 0.5 Da (rCID), respectively. The minimum peptide length was 7 amino acids and maximum of two missed cleavages and strict specificity for trypsin cleavage were required. Carbamidomethylation (Cys) was set as fixed modification, and oxidation (Met), N-acetylation as variable modifications. A 1% false discovery rate (FDR) at the protein and peptide level was required. The scores were calculated as described previously (Cox et al, 2011). The re-quantification and match between runs features were enabled and the relative quantification of the peptides against their SILAC-labeled counterparts was performed by MaxQuant. For protein group quantification unique peptides were used and we required proteins to be quantified with at least two ratio counts. The protein groups file generated by MaxQuant was analyzed in Perseus using the normalized H/L ratios as expression columns. Common contaminants (Cox et al, 2011), reverse and proteins only identified by site were removed. Proteins identified with at least one unique peptide were kept for further analysis. The reported H/L ratio was inverted in spike-in SILAC or reverse SILAC labeling experiments for easier comparison. Ratios and intensities were transformed using $\log_2$ or $\log_{10}$, respectively. Annotations from online databases including the UniProt keywords were added. In SILAC spike-in experiments the ratio of ratios between experimental conditions was calculated.

Primary antibodies, RNAi and primers

Primary antibodies: CCN1/CYR61 (sc-13100), GAPDH (sc-48167) and VE-Cadherin (sc-6458) were from Santa Cruz, phospho-myosin light chain 2 (T18/S19) (3674) and phospho ERK1 (T202/Y204)/ERK 2 (T185/Y-187) (9102) were from Cell Signaling, phospho-FAK (Y397) (700255) from Invitrogen, N-Cadherin (610920), β-Catenin (610153) and Meca32 (550563) from BD biosciences, anti-active-β-Catenin (clone 8E7) (dephosphorylated on S37/T41) (05-665) and Ng2 (MA5320) from Millipore. The N-Cadherin blocking Ab (clone GC-4) (C3865) and human plasma fibronectin (F0895) were purchased from Sigma. CD3 (clone 17A2, 100204), CD19 (clone 6D5, 115506), Ter119 (clone TER119, 116206), NKp46 (clone 29A1.4, 137606), Ly6G (clone 1A8, 127610), Ly6C (clone HK1.4, 128037), CD11b (clone

M1/70, 101259) and CD115 (clone AFS98, 135513) were from Biolegend. For intravital imaging, Alexa Fluor 594 Pecam1 (clone 390, CBL1337) or FITC Pecam1 (clone 390, 102406) from Biolegend was used.

The siRNAs consisted of a pool targeting CCN1 (SI02626421, SI02626428, SI03028655, SI03053477) or CTNNB1 (SI02662478, SI00029743, SI00029750, SI04379655) from Qiagen and a non-targeting pool (D-001810-10-05, GE Healthcare Dharmacon) was used as a control. ShRNAs for CCN1 were from GE Healthcare Dharmacon (V2LHS_271510, V3LHS_330487) or the empty GIPz vector was used as a control. Primers for RT-PCR were as follows (5'-3'): human CCN1 (Fwd- tgcagagctcagtcagagg, Rev- aaatccgggtttctttcaca), human CDH2 (Fwd- gatgaagaaggtggaggagaag, Rev- ccgctttaaggccctcatta), LacZ (Fwd- gaaagctggctacaggaa, Rev- gcagcaacgagacgtca) and housekeeping genes 18S rRNA (Fwd- aggaattgacggaagggcac, Rev- ggacatctaagggcatcaca), GAPDH (Fwd- gtcaccggatttggtcgtattg, Rev- tggaagatggtgatgggattt), and/or ActB (Fwd- tggcaccacaccttctacaat, Rev-tagcaacgtacatggctggg) were used for normalization. In addition the following Taqman probes were used from Integrated DNA Technologies: MelanA assay (Mm.PT.49.16525505), CCN1 assay (Mm.PT.58.9807392.gs), CDH2 assay (Mm.pt.58.9896200) and housekeeping genes GAPDH assay (Mm.PT.39a.1), ActB assay (Mm.PT.47.5885043.g/Mm.PT.58.33257376.gs), and/or Hypoxanthine-guanine phosphoribosyltransferase (Mm.pt.56a.32092191) were used for normalization.

Atomic force microscopy

Atomic force microscopy (AFM) analysis was performed using the Nanowizard 3 AFM model (JPK Instruments). Frozen tissue blocks were cut into 20 µm sections and each section was rehydrated in PBS and thawed at room temperature prior to the AFM analysis. During the measurements, the samples were immersed in PBS containing protease and phosphatase inhibitors (Halt protease and phosphatase inhibitor cocktail, ThermoFisher Scientific). Silicon spheres of 20 µm diameter were attached to tipless monolithic silicon cantilevers with a nominal spring constant of 0.05 N/m (Arrow

TL1, NanoWorld) and calibration was performed by thermal tuning using the simple harmonic oscillator model. Tissues were indented at a 7 µm/sec loading rate with a maximum force of 5 nN and force displacement curves were obtained from mapping 10 µm x 10 µm area fields. To compute the Young's elastic modulus (E), the Hertz model equation for spherical tips was fitted to the force-displacement curves using the JPK Data Processing software (version 4.2, JPK Instruments). An effective indentation of 500 nm was selected and a Poisson's ration of 0.5 was used in the calculation of the Young's elastic modulus.

Intravital Imaging analysis

The time lapse image sequences were imported into FIJI and both channels corrected for fluorescence bleaching using the histogram matching bleaching correction method [https://imagej.net/Bleach_Correction]. Maximum intensity projection images were then generated and the channels recombined. A process of manual inspection was used to count the number of fluorescently labelled B16F10 cells within the field of view and to define cells as attached to the vessels as those cells that remained in physical contact with a vessel for more than 2 time frames (3 min). The ratio of attached to total number of cells in the field of view was then calculated. To quantify cells motility, a parameter was created by calculating the number of cellular events (protrusion extension/retraction and whole cellular body translation) per second. This parameter was then normalized to the total number of cells in the field of view.

Expression and purification of TAT-Cre/HTNC

TAT-Cre construct cloned in pTriEx was expressed with an N-terminal histidine tag in BL21(DE3)pLysS cells (Promega) (Michael Peitz). Protein overexpression was induced by adding isopropyl β-D-thiogalactoside (IPTG) to a final concentration of 250 µM and the cells were harvested 3 hours after induction at 37 ^{o}C. Cell pellets were suspended in lysis buffer (100 mM NaH_2PO_4, 10 mM TrisHCl (pH 8.0), 300 mM NaCl, 10 mM imidazole, 2 mM β-mercaptoethanol, 10 mM phenylmethylsulfonyl

fluoride (PMSF)). Cells were lysed and a two-step purification including a nickel affinity chromatography followed by gel permeation chromatography was performed. The purified protein was concentrated and stored at -80 ^{o}C in the gel permeation buffer (100 mM NaH_2PO_4, 10 mM TrisHCl (pH8.0), 300 mM NaCl, 2 mM β-mercaptoethanol).

Tail vein injection B16F10 cells

For the long term metastasis assay (end point), 14-18 week old C57BL/6 *Ccn1 WT* or *Ccn1 KO^{EC}* mice (6 mice/group) were tail vein injected with 1x10^6 B16F10 melanoma cells in 200 µl of PBS. Mice were humanely culled by cervical dislocation and inflated via the trachea with PBS. Lungs were formalin fixed and paraffin embedded and subjected to haematoxylin and eosin staining to count metastasis (1 sections/lung). For the short term (24h) metastasis assay, 8 week old C57BL/6 *Ccn1 WT* or *Ccn1 KO^{EC}* mice (2 mice/group) were tail vein injected with 1x10^6 GFP$^+$ B16F10 melanoma cells in 200 µl of PBS. Twenty hours after injection, mice were humanely culled by cervical dislocation. Half of each lung was formalin fixed and paraffin embedded. The other half was dissected, minced and digested in RPMI/5% FBS/1 mg/ml Collagenase D (Roche Diagnostics)/10 µg/ml DNase I (Roche Diagnostics) for 45 minutes at 37 °C with agitation; the lung digestion was passed through a 70 µm cell strainer forming single cell suspensions prior to red blood cell (RBC) lysis (8.3 g NH_4Cl, 1.0 g $KHCO_3$, 37.2 mg Na_2EDTA, 1 liter dH_2O, pH 7.2-7.4). After RBC lysis cells were washed with and re-suspended in RPMI/10% FBS/2 mM EDTA and used for flow cytometry analysis.

Flow cytometry analysis

Single cell suspensions were diluted in PBS/1% bovine serum albumin (BSA)/0.05% sodium azide, and live-cells counted in a 1:1 dilution with Trypan blue (Gibco, Thermo Fisher Scientific) using a hemocytometer (Hausser Scientific). Cells were plated at 500,000 cells/well in v-bottom 96 well plates and incubated with a viability dye (Zombie NIR, Biolegend). Cells were washed with PBS/1% BSA/0.05% sodium azide and re-suspended in anti-mouse CD16/32 FcR blocking reagent (TruStain

FcX, Biolegend) followed by incubation with a combination of directly conjugated antibodies for 30 minutes at 4 °C. Cells were washed and re-suspended in PBS/1% BSA/0.05% sodium azide for analysis. Samples were run on the LSRFortessa cell analyzer running FACSDiva software (BD Biosciences) and analyzed using FlowJo software version 10. For the analysis of myeloid cells isolated from C57BL/6 *Rosa26*$^{flSTOP\text{-}tdRFP}$ mice treated or not with HTNC, After RBC lysis cells were washed with and re-suspended in RPMI/10% FBS/2 mM EDTA. For the identification of neutrophils, cells isolated from bone marrow and blood were gated on: FSC-SSC, single cells, live cells, lineage$^-$, Ly6G$^+$, CD11b$^+$, CD115$^-$. Monocytes were gated on: FSC-SSC, single cells, live cells, lineage$^-$, Ly6G$^-$, CD11b$^+$, CD115$^+$, Ly6C$^+$. Lineage$^+$ cells gated on: FSC-SSC, single cells, live cells, lineage$^+$.

IHC and in situ hybridization quantification

Immunohistochemistry staining was performed on 4 µm thick sections of formalin-fixed, paraffin-embedded tissue following standard protocols. The antigen retrieval was performed with Sodium Citrate at pH 6.0 for 2 minutes at 98 °C using a Dako Pre-Treatment module. Sections were stained using anti-Pecam1 (ab28365, Abcam), anti-RFP (600401-379; Tebu), anti-GFP (2555; Cell Signaling). In situ hybridization was performed on sections from tissue which were formalin fixed over night at room temperature and paraffin embedded. In situ-hybridisation detection for Ccn1 (429008; Advanced Cell Diagnostics) and Cdh2 (489578; Advanced Cell Diagnostics) mRNA was performed on formalin fixed paraffin embedded sections using RNAscope 2.5 LS (Brown) detection kit (322100; Advanced Cell Diagnostics) on a Leica Bond Rx autostainer strictly according to the manufacturer's instructions. Quantification was performed using Slidepath software (LeicaBiosystems). Regions of interest were selected and color thresholds set to discern and quantify the areas of red/brown positive staining within the regions of interest. For Cdh2 mRNA quantification in the lung, nine 500 μm^2 regions of interest (regions with club cells and smooth muscle cells were excluded) were quantified by manual counting of positive staining and normalized by cell content (haematoxylin staining).

β-Catenin Luciferase activity assay

Super 16x TOPFlash vector containing firefly luciferase downstream of a series of β-Catenin target TCF/LEF site repeats (16 in total, constructs kindly provided by Professor Mike Olson) were transfected in HUVECs either before seeding on PAGs or together with siRNA, and treated for 24h. Cells were collected in cell culture lysis reagent provided with the Luciferase assay system (Promega E1500) and vortexed before centrifugation and the supernatants were assayed for luciferase activity using the luciferin substrate following the manufacturer's instructions. Luciferase activity was measured using a GloMax 96 well Fluorescent Scanner (Promega) and normalized to total protein content.

Cancer cell adhesion to EC monolayer

HUVECs or HMVECs were seeded at complete confluency for 24h in normal growth media. Then cells were washed briefly in PBS (with Ca/Mg), and the medium replaced with M199 (10% FBS) to prevent EC stimulation. Cancer cells were labeled in serum-free growth media using CellTracker orange CMTMR dye (C2927, LifeTechnologies) or CellTracker green CMFDA dye (C2925) for 30 mins before harvesting and washing to remove excess dye. Labeled cancer cells were resuspended in M199 and an equal quantity added to each well and mixed gently. After 45 mins incubation at 37°C, unbound cells were washed away with PBS (with Ca/Mg) and fixed with paraformaldehyde. ECs were stained (not shown) to ensure an intact monolayer, in addition to DAPI staining, before imaging using a Zeiss 710 confocal microscope. For the 24h adhesion assay, an Operetta High-Content Imaging System was used and the images were analyzed with Columbus software.

Transendothelial migration and permeability assay

$2x10^5$ HMVEC transiently transfected with siCCN1 or siCTL were plated onto fibronectin (Sigma)-coated 24 well transwell inserts (pore size 8 µm, Corning) and cultured for 24h. The integrity of the endothelium monolayer was evaluated by adding 70 kDa FITC dextran (Sigma) on top of each

transwell insert. The fluorescence of the medium in bottom well was measured after 1 hour by using Safire2 photometer microplate reader (TECAN). Empty transwell insert was used as control.

For the transendothelial migration assay, 1x10^5 PC3 TEM 4-18 cells pre-labelled for 30 minutes with the Green CMFDA CellTracker^{TM} (Invitrogen) were plated onto the HMVEC monolayer. After 20 hours, the upper side of the transwell insert was cleaned with a cotton swab to remove cells that did not migrate through the transwell insert. The HMVEC monolayer and PC3 cells that migrated to the lower side of the transwell inserts were fixed with 4% paraformaldehyde, 20 min at room temperature. Cells were permeabilized with Triton X-100 for 30 min at room temperature and stained with anti-VE-Cadherin (Santa Cruz Biotechnologies) followed by Alexa Fluor 555 and DAPI to visualize the endothelium. The transwell insert membranes were cut with a thin scalpel and placed on a slide with the lower side of the membrane up. Images were acquired using Zeiss 880 Airyscan with Fast module (five fields, 10x). Images were analyzed using IMARIS software version 8.3.1 and 8.4.1.

Western blot

Cell lysates were collected in 2% SDS, 100 mM TrisHCl pH 7.6, sonicated and centrifuged before the supernatant was collected and quantified using the Optiblot Bradford reagent (Abcam ab119216). Equal quantity of protein was mixed in loading buffer containing DTT and boiled for 5 mins. Samples were run in 4-12% gradient NuPAGE Novex Bis-Tris gels. Proteins were transferred to activated polyvinylidene fluoride membrane using a wet electroblotting system operated at 100 V constant for 1 h in transfer buffer (Life technologies, NP0006-1). Membranes were blocked overnight in 5 % milk or 3 % BSA at 4°C, before primary antibody incubation, and subsequent secondary antibodies. Western blots were analyzed with LI-COR scanning (Odyssey CLx Scanner) or MyECL Imager (ThermoFisher Scientific) or enhanced chemiluminescence using x-ray film and scanned using a

calibrated densitometer (GS-800, Bio-Rad). Western blot were quantified using Image Studio Lite software.

Immunofluorescence staining

Cells were fixed in 4% paraformaldehyde and permeabilized in 0.02% saponin for 30mins. Primary antibodies were incubated for 1h at a dilution of 1:100 in 3% BSA, 0.02% saponin. Fluorescently labeled secondary antibodies were incubated for 45 mins in the same dilution buffer before counter staining with DAPI and mounting on glass slides. Images were acquired using a Zeiss 710 confocal microscope or with Operetta High-Content Imaging System.

Reid et al Appendix Figure S1

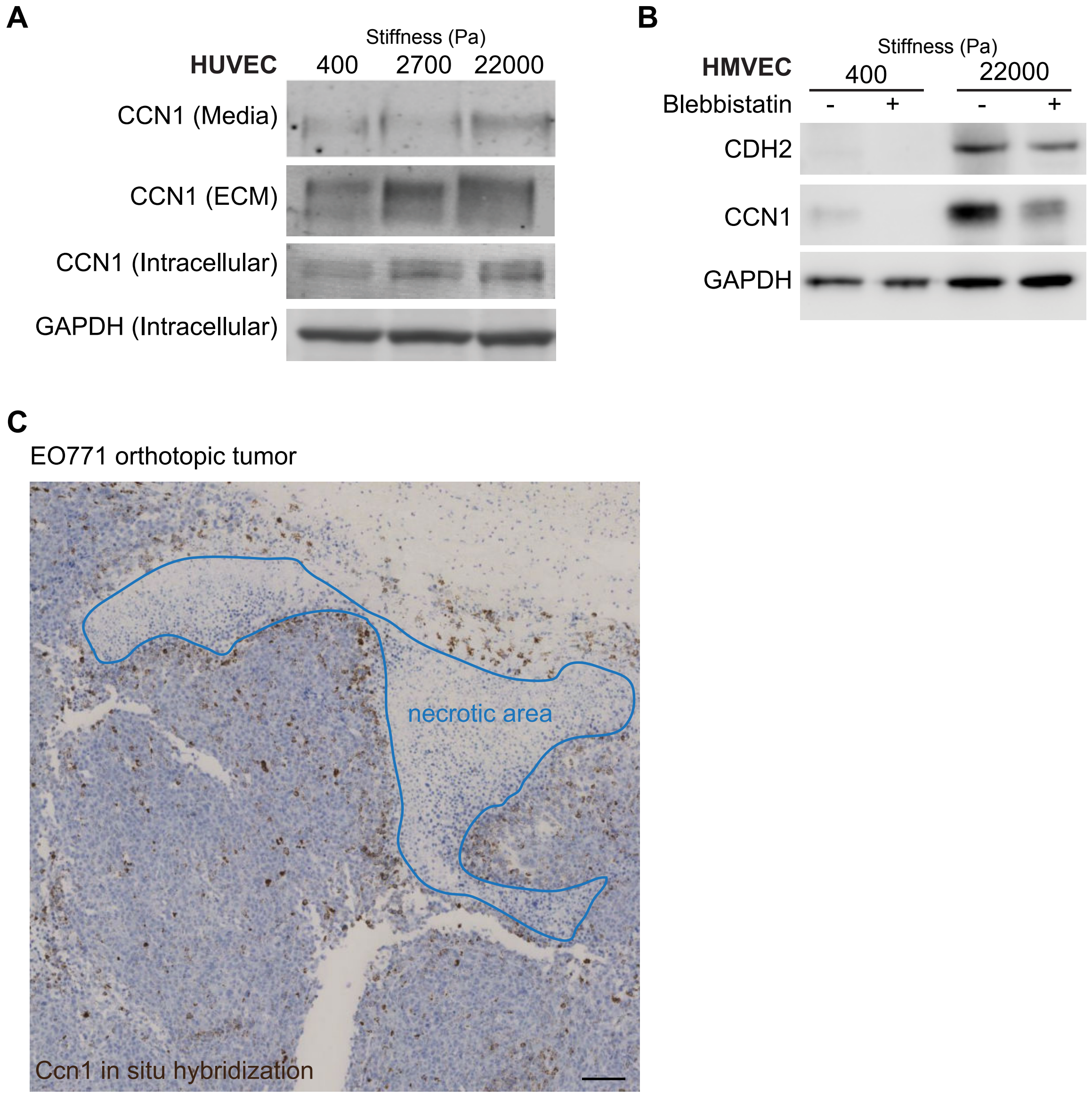

Reid et al Appendix Figure S2

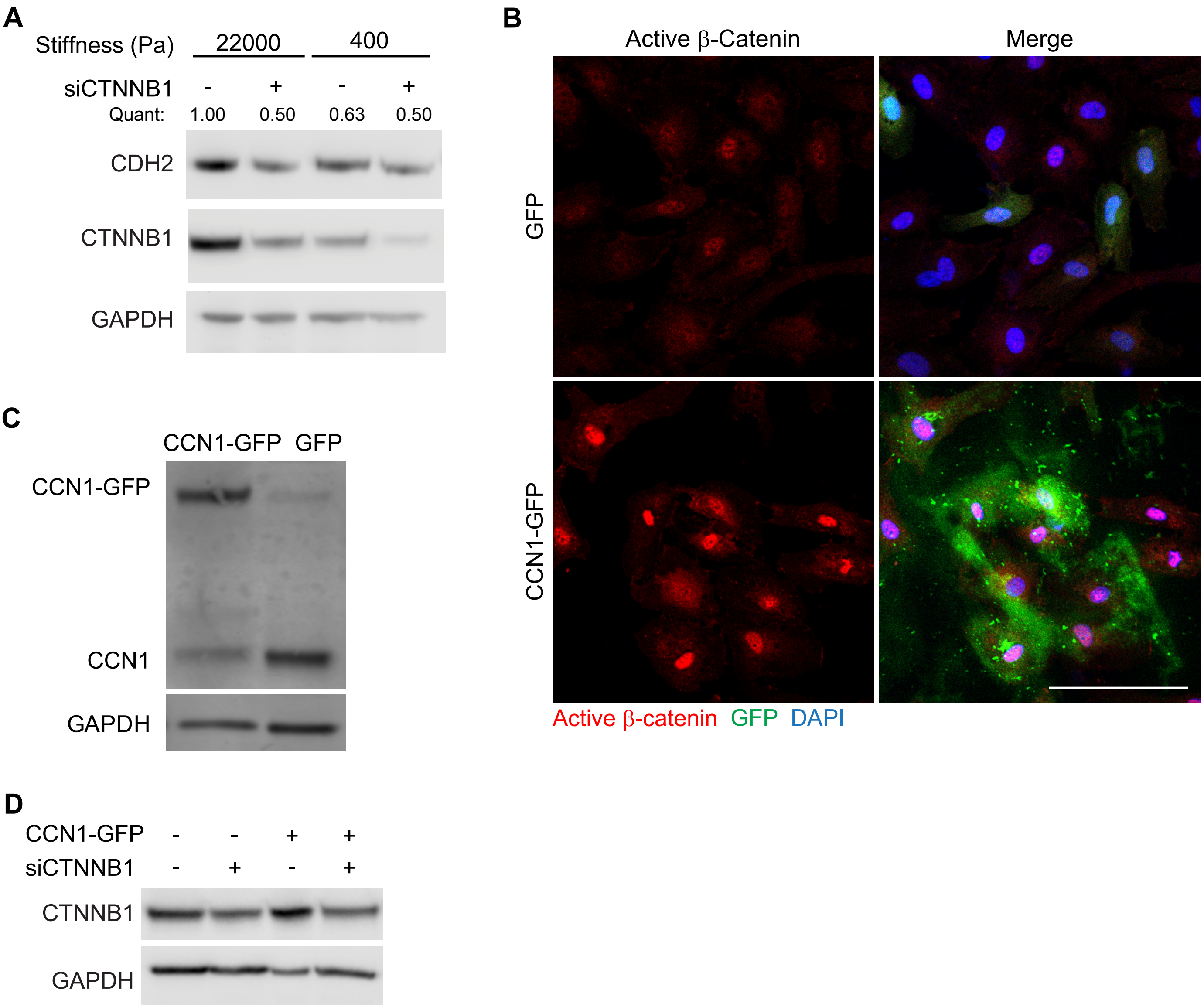

Reid et al Appendix Figure S3

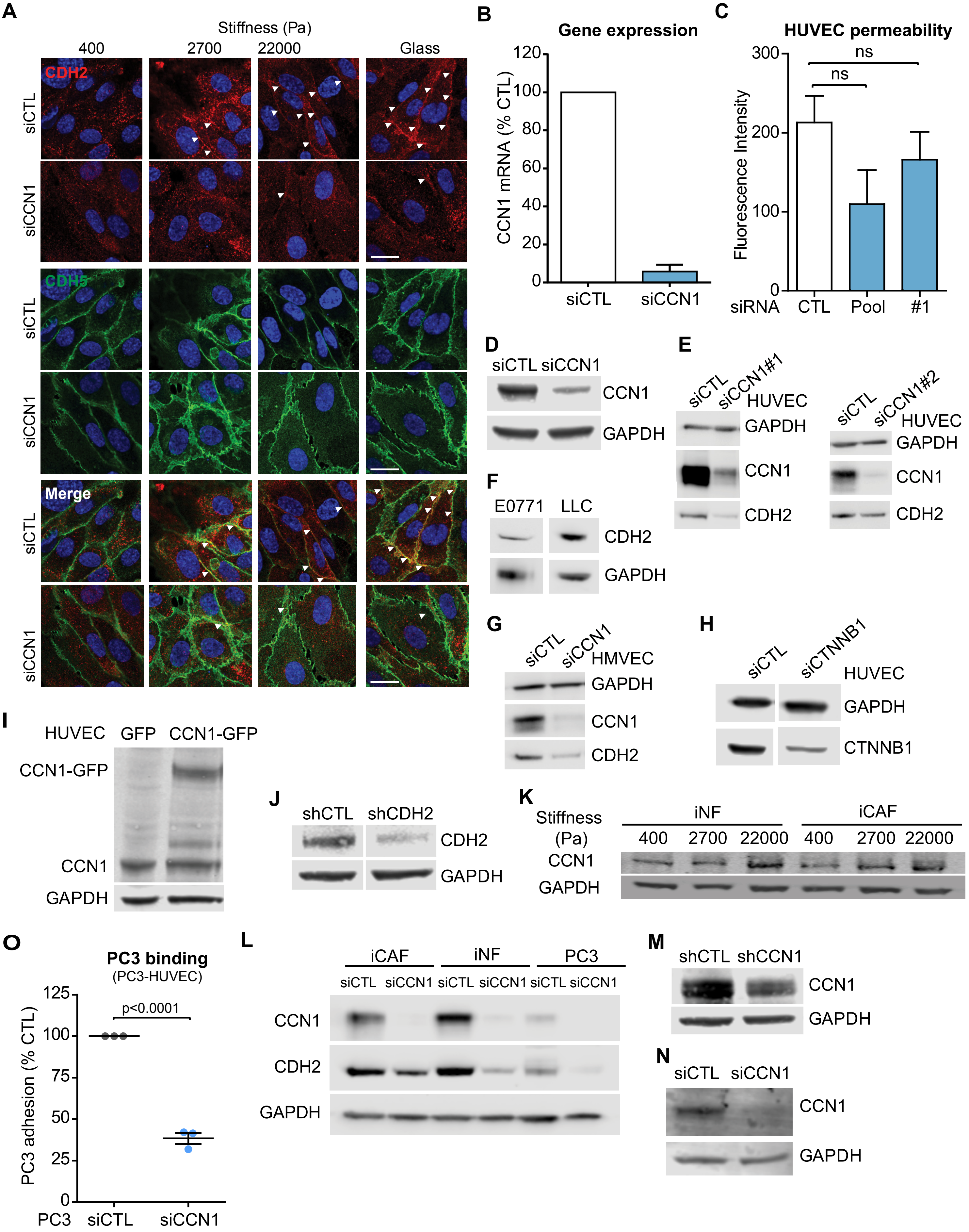

Reid et al. Appendix Figure S4

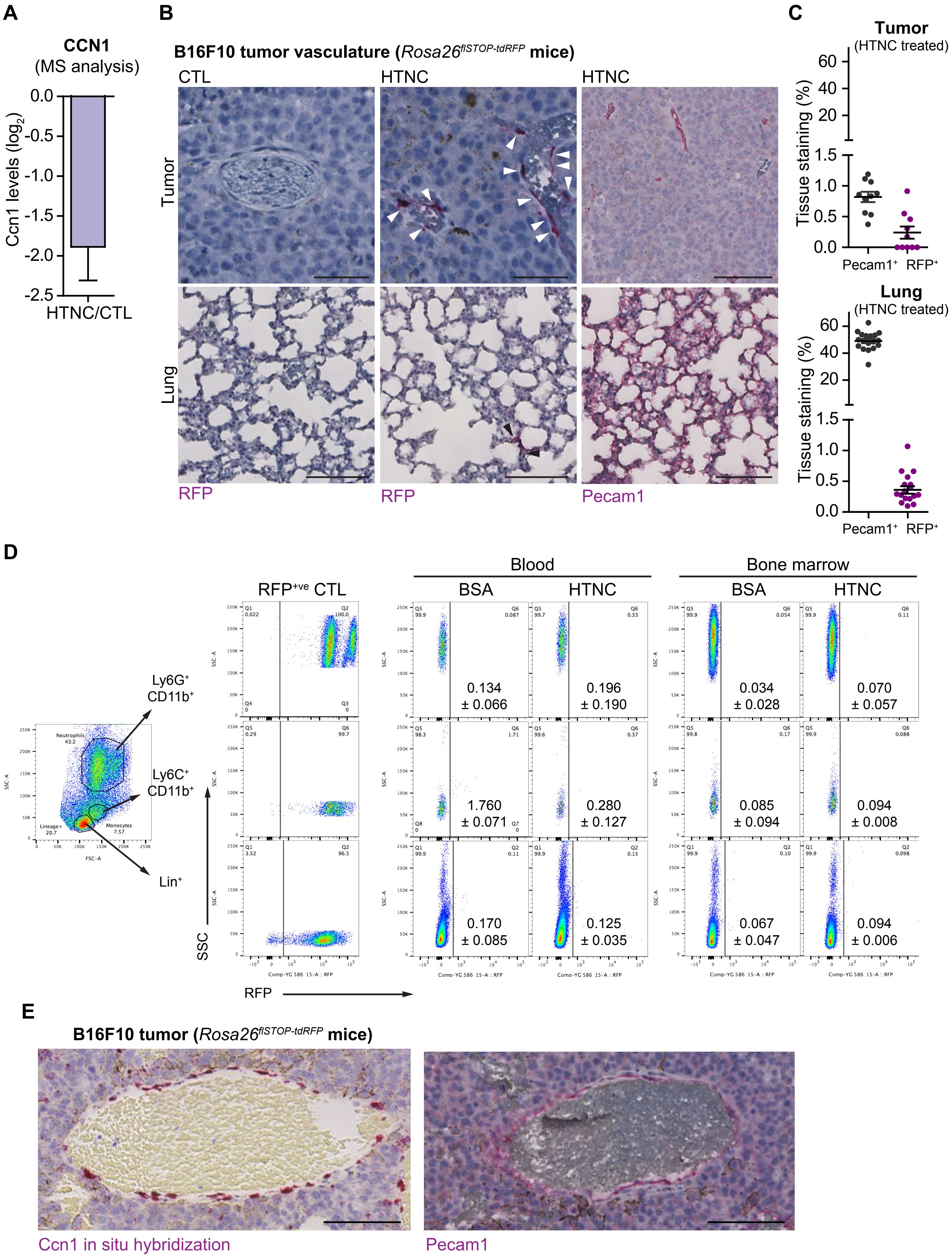

Reid et al Appendix Figure S5

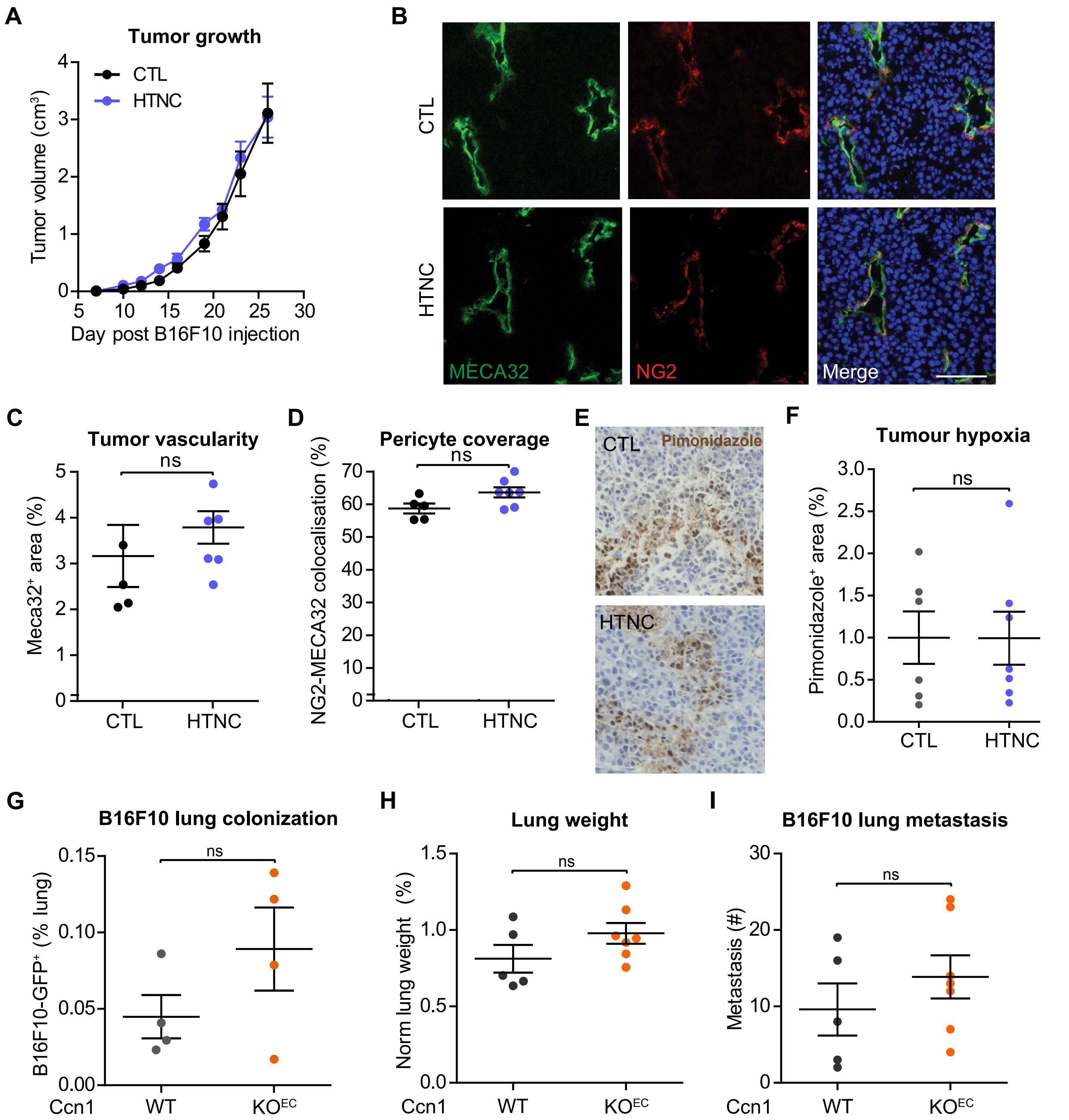

Reid et al Appendix Figure S6

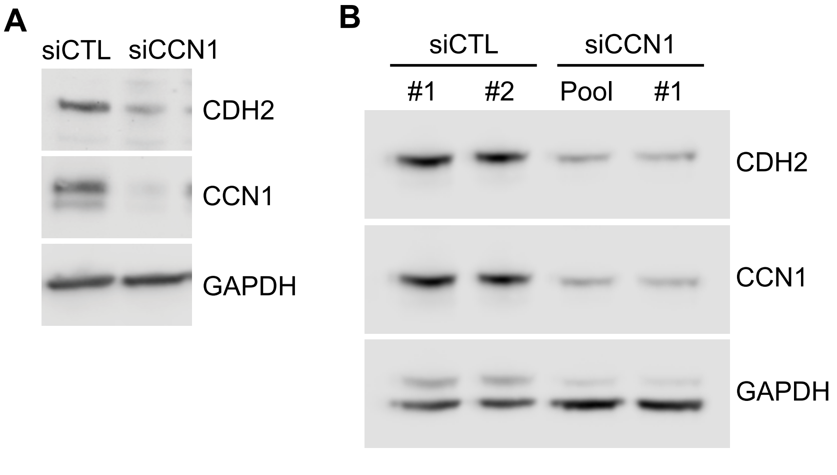

Supplement: Supplementary file 1 — Appendix [file EMBJ-36-2373-s001.pdf]
