# Supplementary material for: Tumor matrix stiffness promotes metastatic cancer cell interaction with the endothelium
Source: EMBO J. 2017 Jul 10;36(16):2373–89. doi: 10.15252/embj.201694912 (PMC5556271; doi:10.15252/embj.201694912)
Supplement: Supplementary file 2 — Expanded View Figures PDF [file EMBJ-36-2373-s002.pdf]

Expanded View Figures

Figure EV1. Stiffness-regulated changes in HUVEC proteome.

A Scatter plot of the 5,461 HUVEC proteins identified and with a calculated ratio between low stiffness and high stiffness in replicate experiments (forward, Fw, and reverse, Rev, experiment) measured by MS analysis. The colors represent the density distribution (0 = 0% and 100 = 100%) of the SILAC ratios calculated in the Fw and Rev experiments (Perseus analysis). The colored panels indicate the subset of proteins that were upregulated (red) and downregulated (blue) in both replicate experiments. Outliers (likely contaminants) in the upper left panel have been excluded to allow better visualization of the data.

B Representation of the physical and functional interactions among the stiffness-regulated proteins in HUVECs, as measured by STRING and visualized by Cytoscape. Red = upregulated with stiffness; blue = downregulated with stiffness.

C Pathway enrichment analysis of the regulated proteins, as calculated by STRING.

D Example of cell–cell receptors that were found upregulated by stiffness in HUVECs. The plot reports the calculated SILAC ratio in the Fw and Rev experiments. Bars represent mean ± SEM.

© 2017 Cancer Research UK Beatson Institute

A

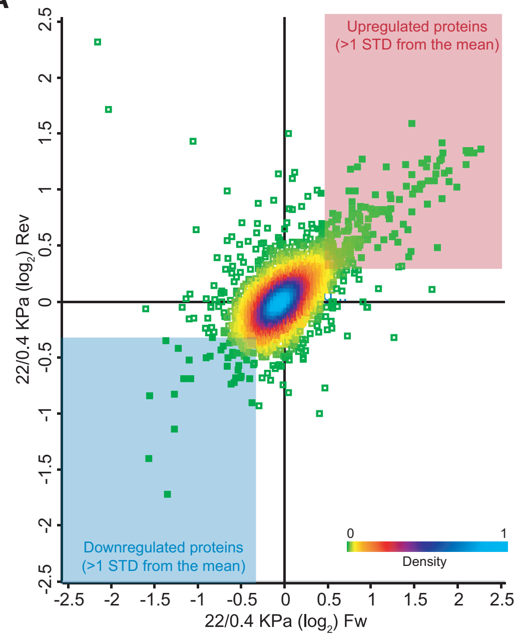

C

Pathway ID (STRING)	Pathway description	Observed gene count	FDR
4514	Cell adhesion molecules (CAMs)	11	2.01E-04
240	Pyrimidine metabolism	9	4.33E-04
1100	Metabolic pathways	32	7.70E-04
4110	Cell cycle	9	8.29E-04
4115	p53 signaling pathway	7	8.29E-04
3030	DNA replication	5	2.26E-03
5166	HTLV-I infection	12	2.26E-03
100	Steroid biosynthesis	4	2.91E-03
3410	Base excision repair	4	1.93E-02
5219	Bladder cancer	4	2.71E-02

D

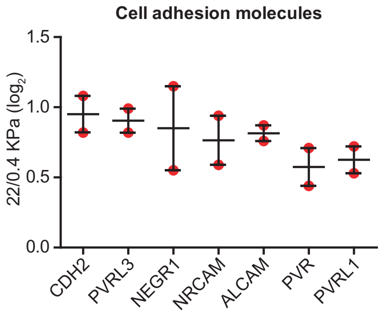

B

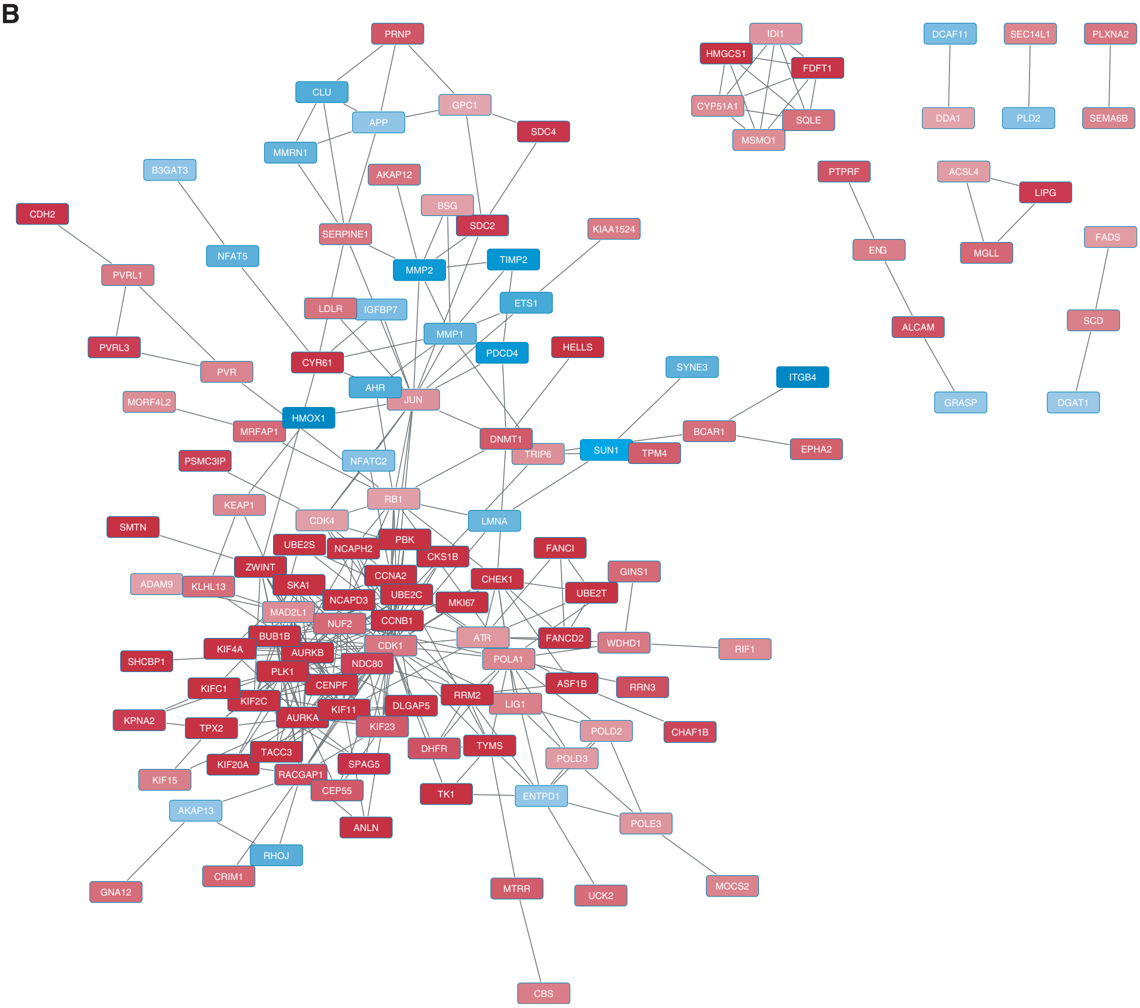

Figure EV1.

Figure EV2. CCN1 controls N-cadherin levels in endothelial cells.

A, B Spread area (B) and proliferation (A) of HUVECs cultured on FN-coated PAGs of indicated stiffnesses were not altered following CCN1 knockdown. Significance according to two-tailed unpaired *t*-test. Data are represented as mean ± SEM. For panel (B), *n* = fields assessed from one representative experiment of three biological replicates; siCTL: *n* = 25 (400, 2,700 Pa), *n* = 30 (22,000 Pa); siCCN1: *n* = 19 (400, 22,000 Pa), *n* = 21 (2,700 Pa). For panel (A) *n* = 10 fields per stiffness from 3 replicate experiments.

C Representative Western blot for CCN1 shows knockdown efficiency in HUVECs used in panels (A and B).

D, E Representative Western blot analysis (D) and quantification (E) showing that in HMVECs, stiffness induces CCN1 and N-cadherin (CDH2) levels and that silencing of CCN1 with a pool of siRNAs reduces N-cadherin levels. CCN1 and N-cadherin quantification based on Image Studio Lite software. Bars represent mean ± SEM (*n* = 3 replicate experiments).

F, G Representative Western blot analysis (F) and quantification (G) showing that in HUVECs, stiffness induces CCN1 and N-cadherin levels and that silencing of CCN1 with a single siRNA reduces N-cadherin protein levels. CCN1 and N-cadherin levels were normalized by GAPDH, which was used as a loading control. CCN1 and N-cadherin quantification based on Image Studio Lite software. Bars represent mean ± SEM (*n* = 3 replicate experiments). Same Western blot shown in Fig 4A.

H Representative Western blot analysis of HUVECs transiently transfected with CCN1-GFP vector and cultured for 24 h showing that GFP-tagged CCN1 is abundantly expressed in whole-cell lysates and secreted in the media at the expected molecular weight (42 kDa for CCN1 + 28 kDa for GFP = 70 kDa).

I Immunofluorescence staining showing that the localization of CCN1-GFP mirrors that of endogenous CCN1 diffusely in the matrix. Cells from a control transfection were stained for CCN1 (green), N-cadherin (red), and DAPI, while CCN1-GFP-overexpressing HUVECs were stained with N-cadherin (red) and DAPI only. Scale bar = 50 μm.

Source data are available online for this figure.

 © 2017 Cancer Research UK Beatson Institute

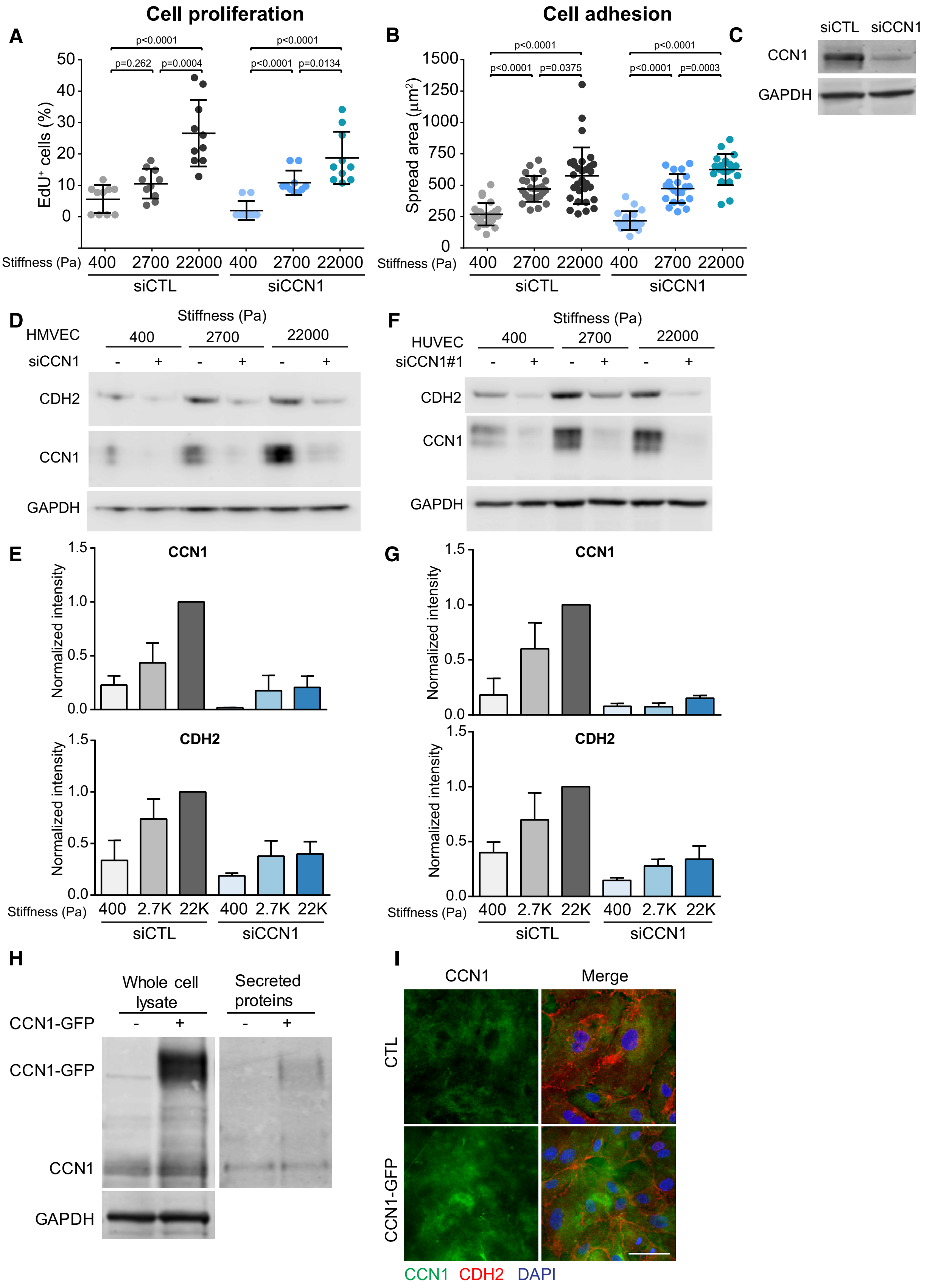
Cell proliferation
A
p<0.0001
p=0.262 p=0.0004
p<0.0001
p<0.0001 p=0.0134
EdU⁺ cells (%)
50
40
30
20
10
0
Stiffness (Pa) 400 2700 22000 400 2700 22000
siCTL siCCN1
Cell adhesion
B
p<0.0001
p<0.0001 p=0.0375
p<0.0001
p<0.0001 p=0.0003
Spread area (µm²)
1500
1250
1000
750
500
250
0
Stiffness (Pa) 400 2700 22000 400 2700 22000
siCTL siCCN1
C
siCTL siCCN1
CCN1
GAPDH
D
HMVEC
Stiffness (Pa)
400 2700 22000
siCCN1 - + - + - +
CDH2
CCN1
GAPDH
F
HUVEC
Stiffness (Pa)
400 2700 22000
siCCN1#1 - + - + - +
CDH2
CCN1
GAPDH
E
CCN1
CDH2
Normalized intensity
1.5
1.0
0.5
0.0
Stiffness (Pa) 400 2.7K 22K 400 2.7K 22K
siCTL siCCN1
G
CCN1
CDH2
Normalized intensity
1.5
1.0
0.5
0.0
Stiffness (Pa) 400 2.7K 22K 400 2.7K 22K
siCTL siCCN1
H
Whole cell lysate
Secreted proteins
CCN1-GFP - + - +
CCN1-GFP
CCN1
GAPDH
I
CCN1
Merge
CTL
CCN1-GFP
CCN1 CDH2 DAPI

Figure EV2.

Figure EV3. Efficient KO of Ccn1 in *Ccn1 KO^{EC}* mice.

A The gene locus of *Ccn1$^{fl/fl}$* mice showing the LacZ reporter gene activation once recombination has occurred.
B Quantification of Ccn1 expression (*in situ* hybridization with RNAscope), showing that Ccn1 is expressed in the lung of *Ccn1 WT* mice, and the efficient depletion of Ccn1 expression in the lung of Ccn1 endothelial-specific conditional KO mice (*Ccn1 KO^{EC}*). *N* = 4 mice assessed per group.
C Representative staining of lung sections quantified in (B). Scale bar = 100 μm.
D Quantification of the immunostaining for Pecam1 in the lung of *Ccn1 WT* and *Ccn1 KO^{EC}* mice showing the absence of significant differences in the total lung vasculature upon depletion of Ccn1 in the endothelium. *N* = 7 (*Ccn1 WT*) and 8 (*Ccn1 KO^{EC}*) mice assessed.
E Representative staining of lung sections quantified in (D). Scale bar = 100 μm.
F Quantification of Ccn1 expression (*in situ* hybridization with RNAscope) showing that Ccn1 is expressed in the ear of *Ccn1 WT* mice and the efficient depletion of Ccn1 expression in the ear of *Ccn1 KO^{EC}* mice. *N* = 4 mice assessed per group.
G Quantification of N-cadherin (Cdh2) expression (*in situ* hybridization with RNAscope; total staining normalized by cell content) showing that depletion of Ccn1 in the endothelium decreases the levels of N-cadherin in the lungs (*n* = 45 (*Ccn1 WT*) and 54 (*Ccn1 KO^{EC}*) fields assessed from 5 female mice for *Ccn1 WT* and 6 female mice for *Ccn1 KO^{EC}*).

Data information: Bars represent mean ± SEM. *P*-values based on two-tailed unpaired *t*-test.

© 2017 Cancer Research UK Beatson Institute

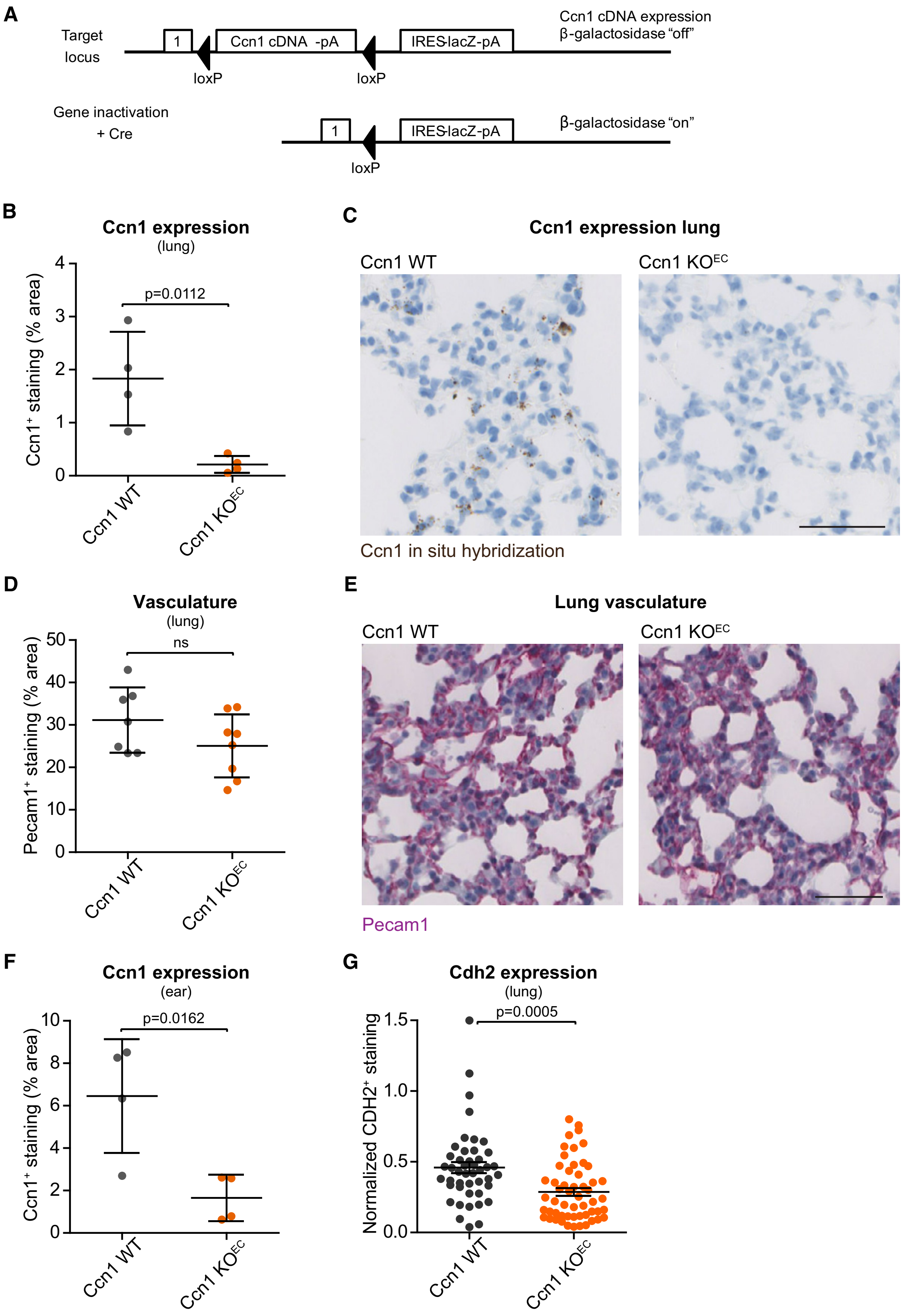

Figure EV3.

© 2017 Cancer Research UK Beatson Institute
