# Supplementary material for: Tumor matrix stiffness promotes metastatic cancer cell interaction with the endothelium
Source: EMBO J. 2017 Jul 10;36(16):2373–89. doi: 10.15252/embj.201694912 (PMC5556271; doi:10.15252/embj.201694912)
Supplement: Supplementary file 4 — Movie EV2 [file EMBJ-36-2373-s004.zip › EMBOJ-2016-94912R_MovieEV2/README_MovieEV2.docx]

## **EMBOJ-2016-94912R_MovieEV2.zip. Intravital imaging of B16F10 cancer cells (red) intradermally injected in the ear of Ccn1 WT^EC^ mouse and interacting with blood vessel (green).**
