# Supplementary figures and images for: Tumor matrix stiffness promotes metastatic cancer cell interaction with the endothelium
Source: EMBO J. 2017 Jul 10;36(16):2373–89. doi: 10.15252/embj.201694912 (PMC5556271; doi:10.15252/embj.201694912)

Unprocessed western blot in Figure S2C

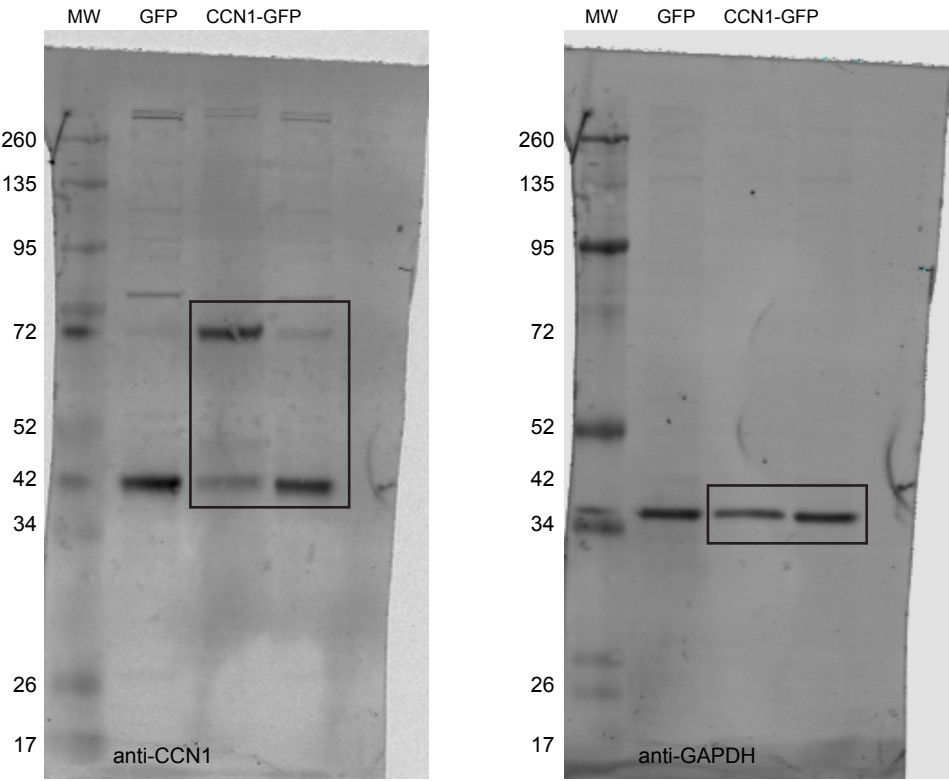

Supplement: Supplementary file 8 — Source Data for Expanded View and Appendix [file EMBJ-36-2373-s010.zip › 94912_Sourcedata_AppendixfigS2C/94912_SourceDataForAppendixFigureS2C.pdf]
